# Assessment of the Wii Basic Balance Test in measuring postural deficits post-concussion

**Published:** 2016-12-12

**Authors:** Nicholas G. Murray, Eileen Fernandez, Anthony P. Salvatore, Rebecca J. Reed-Jones

**Affiliations:** 1 *School of Health and Kinesiology, College of Health Sciences, Georgia Southern University, Georgia, United States*; 2 *Speech Language Pathology Program, College of Health Sciences, University of Texas at El Paso, Texas, United States*; 3 *Department of Applied Human Sciences, Faculty of Science, University of Prince Edward Island, Charlottetown, Canada*

**Keywords:** Wii Fit, postural control, head injury, traumatic brain injury, motor control

## Abstract

**Background and Aim:** To evaluate the Wii Basic Balance Test (WBBT), as a tool for detecting postural instability in athletes with concussions.

**Methods:** Seventy-nine healthy physically active controls (NORM) (mean age 21.23 ± 1.78), and fifty-six athletes with concussions (CONC) (mean age 19.39 ± 2.145) participated in this study. All participants performed the Wii Basic Balance Test, which requires the participants to shift weight mediolaterally to maintain a red bar within a blue area denoted on the screen for three seconds during set levels of difficulty. CONC were included in the study within 24-48 hours of the initial concussion injury. Seven one-way ANOVAs assessed differences for each (1) Mean total number of WBBT Levels completed (TL), (2) Mean total seconds to complete all WBBT Level (TT), (3) Time to complete Level 1 (LI), (4) Time to complete Level 2 (L2), (5) Time to complete Level 3 (L3), (6) Time to complete Level 4 (L4), (7) Time to complete Level 5 (L5).

**Results:** CONC completed significantly fewer Levels of the WBBT (p=0.032) when compared to NORM. Athletes with Concussions took a significantly longer time to complete LI (p=0.002) when compared to CONC. Post-hoc Chi-Square analysis determined a significantly greater (p=0.015) proportion (39%) of CONC successfully completed WBBT L5 when compared to the proportion (19%) of CONC. Follow up ROC curves revealed an Sn = 0.392 and an Sp = 0.821 for TL with a cutoff value of 4 levels, Sn=0.875 and an Sp = 0.253 for LI with a cutoff value of 4.4 seconds, and Sn = 0.804 and an Sp = 0.392 for those who successfully completed the WBBT L5.

**Conclusions:** WBBT could be a low cost object method of assessing postural instability within 24-48 hours post-concussion.

**Relevance for patients:** This data could provide health providers with an alternative method to measure the presence of postural instability post-concussion injury.

## 1. Introduction

Postural control or stability issues are a cardinal sign of a concussion injury [1-4]. Multiple methods exist to assess the extent or presence/absence of a postural dysfunction postconcussion [3,5]. The most common methods for assessing postural stability are the Balance Error Scoring System (BESS) [6], the Romberg Test [3], the Sensory Organization Test (SOT) [7] in combination with the NeruoCom or other force platform technology, and force platform measurements that monitor quiet upright stance [8]. While the BESS and the Romberg Test are considered low technology and are easy to administer, they rely heavily upon trained rater interpretations and lack objectivity [3,5]. The SOT and other force platform measurements require expensive technology, can be difficult to administer and interpret, yet they contain minimal subjectivity [3,5]. All of these methods demonstrate postural instability at 24-48 hours post- concussion and have varying reliability and validity evidence to support their use [3,5]. However, recent technology has been developed that could provide both affordable and objective alternatives to measure postural stability post-concussion.

The Nintendo Wii Fit (Nintendo Co., Ltd., Kyoto, Japan) has progressed from being exclusively an entertainment system to a system used more frequently in research and rehabilitation settings. The Wii Balance Board, that drives or controls the game, provides comparable and reliable force data (center of pressure data) when compared to research-grade force platforms [9,10]. This postural data has been used to develop a low cost and portable assessment tool, that show promise in the detection and monitoring of postural instability [11]. A recent study suggested that in a group of healthy collegiate athletes during play of a pre-loaded Wii Fit game, quiet standing in single and double leg for 30 seconds, was found to be reliable and valid when compared to the BESS performed on a firm surface [12]. Rehabilitation modalities and techniques using a pre-loaded Wii Fit game and/or a custom created game that involves the Wii Balance Board suggests increased postural stability and decreased fear of falling among older adults [11,13,14]. Research has indicated a significant relationship with visual processing speed, a determinant of mobility, and the Wii Fit game, the Basic Balance Test (WBBT) in older adults, yet did not relate to standard fitness or mobility assessments [15].

These promising results could be explained by the Wii Fit’s ability to tap into unique motor strategies that rely heavily on visual fields to accomplish the presented game task. These types of motor strategies are typically observed during sport activity and functional mobility [16]. One such game, WBBT, requires that individuals maintain upright stance while simultaneously reorienting one’s Center of Mass (COM) in the medial/lateral directions in responses to the increasingly more visual and environmental demands of the game [15]. These movements incorporate a mixture of static and dynamic postural control tasks. This combination of dynamic movements of the COM and maintenance of static upright stance could effectively challenge the motor control system and more accurately replicate functional postural stability. Reed-Jones et al. in 2012 found that among older adults, a faster visual processing speed related to how fast one completed the levels of play on the Wii Fit Basic Balance Test [15]. Visual processing speed is the ability to quickly scan one’s environment without head movement [15]. Impaired visual processing speed in older adults is associated with functional mobility issues [17]. These findings could indicate that certain Wii Fit games like the WBBT provide an environmentally relevant postural task that functionally stresses the postural control system in response to the visual context of games [15,18]. No research exists using a Wii Fit game, a custom analogous version of a Wii Fit game, or involving the use of the Wii Balance Board to detect postural impairment in a post-concussion population.

The purpose of this study is to (1) provide normative data of the WBBT in healthy young adults, (2) compare the performance of athletes with concussions to healthy young adults on the WBBT (3) provide sensitivity and specificity values of the WBBT. The aim of this study is to evaluate the WBBT as a potential tool for detecting postural instability in athletes with concussions. It is hypothesized that athletes with concussions will take a longer time to complete the WBBT, complete less levels of the WBBT, and will spend a greater amount of time on each level of the WBBT when compared to the healthy controls.

## 2. Materials and Methods

### 2.1. Participants

Seventy-nine healthy physically active controls (NORM) (mean age 21.23 ± 1.78), and fifty-six athletes with concussions (CONC) (mean age 19.39 ± 2.145) participated in this study. All participants were aged 18-25 years, physically active for greater than 10 hours per week (prior to the concussion injury), and free of any musculoskeletal and/or neuromuscular injury beyond the documented concussion injury. In addition, no participants had a history of meningitis, hydrocephalus, hydranencephaly, balance disorders, seizures, and/or any form of attention deficit disorders as determined by self-report. Five CONC self-reported a total of two previous concussions and a single CONC self-reported three prior concussions. All other CONC had no self-reported history of concussion. NORM participants were excluded from participation in the study if they had a history of concussion as determined by self-report. All CONC had a confirmed concussion by the head athletic trainer at their school of residence and/or medical doctor on staff. CONC were referred by a participating athletic trainer or doctor and participated in the study within 24-48 hours post-concussion. All participants gave their informed written consent to participate in the study. The University Institutional Review Board approved this study prior to any data collection.

### 2.2. Procedures

**Figure 1.**
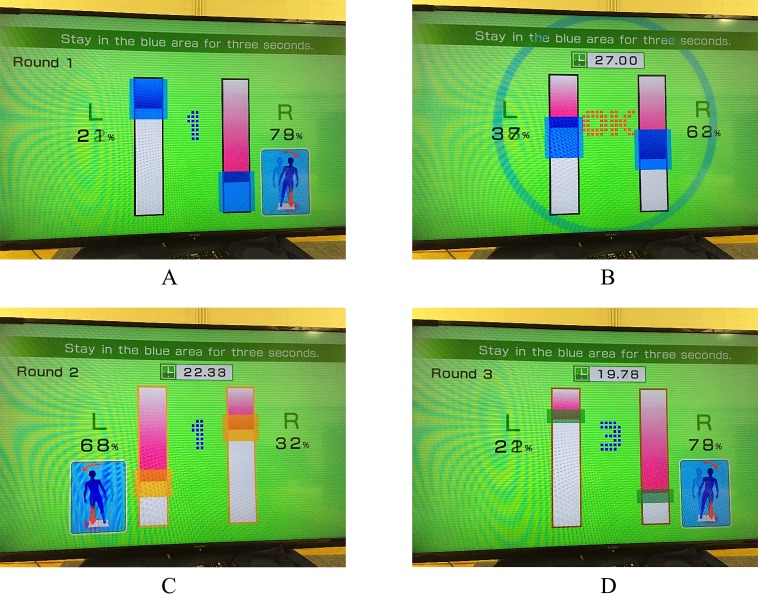
The Wii Fit Basic Balance Test (WBBT). During play, the individual will shift their center of mass mediolaterally to place a certain amount of pressure onto each limb and hold that position for three seconds. When holding the position the individual must move the red vertical bars into the blue horizontal bars and not move the red bars outside of that area. If the area red vertical bars are not maintained for three full seconds inside the blue horizontal bars, the three-second timer starts again. The total time allotted for the game is 30 seconds. The game difficulty increases from the first Trial until the third Trial as the width of the target area decreases. Caption A indicates Level 1 of the WBBT. Caption B refers to the visual feedback of the game upon completion of the three-second timer. Caption C demonstrates Level 2 and the decreased width of the target bars. In addition, is shows the visual feedback (alteration from blue to yellow area) that the participants red horizontal bars are within the appropriate area has successfully accomplished the trial. Caption D refers to WBBT Level 3 with the three-second timer just beginning after successful movement into the target bars. The shift from blue to green within the target area is visual feedback that the three second timer will begin counting down.

Athletes with Concussions performed the WBBT after completion of a computerized cognitive test that is not reported in this publication. A rest period of 15 minutes was given to each CONC in order to limit fatigue and minimize further testing bias. The WBBT used the Wii Balance Board (WBB) which was sensitive to shifts in weight anteroposteriorly and mediolaterally to direct one’s center of pressure to adjust to a set target area on a visual display (Figure 1. The game requires the participants to shift weight to the left and right to maintain two horizontal red bars within a colored area denoted on the screen for three seconds. Upon correct placement of the horizontal bar, a timer will commences starting at three seconds. The blue area will shift to green to indicate correct placement and then will turn yellow upon completion of the Level. The WBBT has five Levels of difficulty that individuals must complete within the thirty seconds allotted for the entire test. The final score is how many levels the participant was able to complete successfully, along with the time it took to complete each Level and a total time to complete the test. At each of the five Levels of difficulty (Levels 1-5); the task becomes more demanding by shrinking the blue area that the red bar must fit (See Figure 1). This red bar must be maintained for three seconds during each Level. If the red bar moves out of the blue bar without completing the three-second-time frame, the three-second timer is reset, and the participant must try again at that Level. Participants cannot proceed to the next level of difficulty of play until they have successfully completed that level. Participants can fail to achieve all Levels on the WBBT as the total time to complete the assessment is thirty seconds for all five levels. As such, a fail to complete at each level is dependent upon the time it takes them to complete each level. For example, if a participant takes 30 seconds to complete level 1 and 2, they fail to complete levels 3-5. In short, at successive Levels the target gets smaller and thus more demanding. All participants were given an untimed practice test to familiarize them with the game.

### 2.3. Statistical design

Seven one-way ANOVAs assessed differences for each dependent measure: (1) Mean total number of WBBT Levels completed (TL), (2) Mean total seconds to complete all WBBT Level (TT), (3) Time to complete Level 1 (LI), (4) Time to complete Level 2 (L2), (5) Time to complete Level 3 (L3), (6) Time to complete Level 4 (L4), (7) Time to complete Level 5 (L5), using SPSS (version 23, IBM, Armonk, New York). The time to complete each Level is denoted in seconds. Post-hoc Chi-Square analyses were conducted to ascertain the association and proportion of total number of participants who successfully completed each WBBT Levels 1-5. In addition, post-hoc Receiver Operator Characteristics (ROC) Curve were assessed to determine the sensitivity (Sn) and specificity (Sp) for dependent variables that met the desired alpha level. An alpha of 0.05 was set a priori with bonferroni corrections for multiple comparisons.

## 3. Results

Of the CONC, 100% (79/79) were able to complete WBBT Levels 1-3, 79% (63/79) were able to complete WBBT Level 4, and 39% (31/79) were able to complete WBBT Level 5 (See Table 2). Of the CONC 100% (56/56) were able to complete WBBT Levels 1-2, 99% (55/56) completed WBBT Level 3, 73% (41/56) completed WBBT Level 4, and 19% (11/56) completed Level WBBT 5 (see Table 1).

**Table 1.** The Number of Levels and average time to complete the Wii Basic Balance Test in Healthy Controls and Athletes with Concussions.

Controls (N = 79)	Number of WBBT levels of difficulty	Number of levels completed (%)	Average time to completion of level (seconds)
	1	100	4.42 ± 1.16
	2	100	5.21 ± 1.28
	3	100	6.40 ± 1.86
	4	79	7.71 ± 2.10
	5	39	5.88 ± 1.41
	1	100	5.18 ± 1.56*
Athletes with concussions (N = 56)	2	100	5.66 ± 1.66
	3	99	6.82 ± 2.13
	4	73	7.88 ± 2.36
	5	19†	5.49 ± 0.99

Note: * = Oneway ANOVA significantly different from healthy controls at p < 0.05,

† = Chi-Square significant association with controls on completed WBBT Level 5 when compared to controls at p<0.05. WBBT = Wii Basic

**Figure 2.**
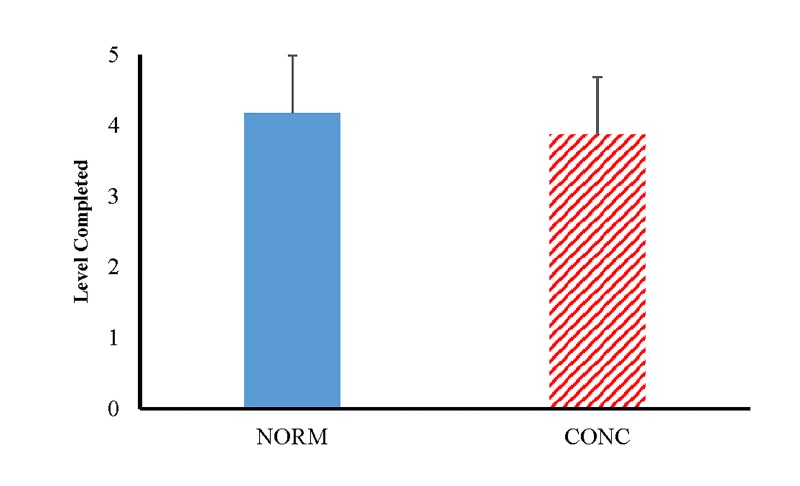
Mean and Standard Deviations of the total number of Levels completed on the Wii Basic Balance Test. Note: NORM = controls and CONC = Athletes with Concussions; *p*-values was determined by a Oneway ANOVA: *p* = 0.032.

Athletes with Concussions completed significantly fewer Levels of the WBBT (F_(133,134)_= 4.704, p=0.032) when compared to CONC. However, TT (F_(189,190)_= 1.047, p=0.308) were not significantly different between groups (CONC = 29.15 seconds, NORM = 28.79 seconds). Athletes with Concussions took a significantly longer time to complete LI (F_(133,134)_= 10.371, p=0.002) when compared to CONC (See Figure 2). No significant differences were observed between groups for L2 (F_(132,133)_= 6.563, p=0.365), L3 (F_(131,132)_= 5.767, p=0.226), L4 (F(102,103)= 0.690, p=0.707), and L5 (F_(40,41_= 0.857, p=0.356), (See Figure 2).

**Figure 3.**
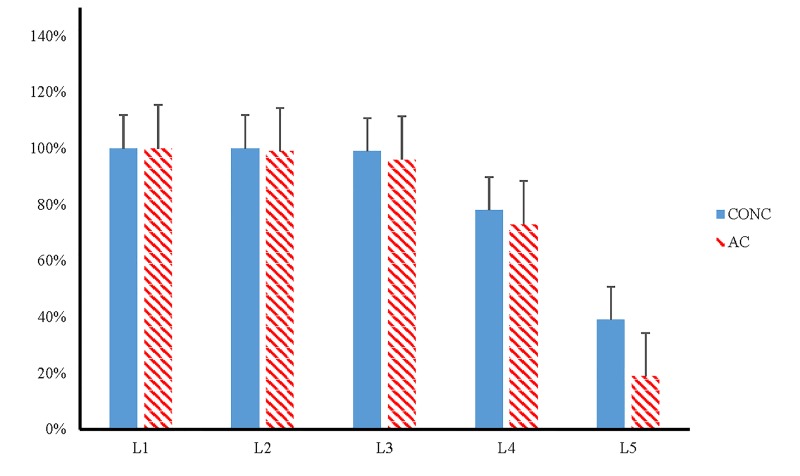
Proportion of Wii Basic Balance Test levels completed by controls and Athletes with Concussions. Note: NORM = control athletes, CONC = Athletes with Concussions; *p*-value was determined by ChiSquare analysis: *p* = 0.015. L1 = Level 1, L2 = Level 2, L3 = Level 3, L4 = Level 4, L5 = Level 5.*p* = 0.015.

Post-hoc Chi-Square analysis determined no significant association among the NORM and CONC who completed WBBT L2 *(p =* 0.415), L3 *(p =* 0.091), and L4 *(p =* 0.374). WBBT Level 1 was not analyzed due to all participants in both groups completing it successfully. Among NORM and CONC who successfully completed WBBT L5, a significant association was observed (*p =* 0.015, <D = 0.209). Therefore, a significantly greater proportion (39%) of NORM successfully completed WBBT L5 when compared to the proportion (19%) of CONC (See Figure 3).

Post-hoc ROC curve analysis revealed that TL is fairly accurate in discriminating the true negative rate, those that do not have a concussion, (AUC=0.608, 95% CI: 0.513 to 0.703; p=0.033) with an associated Sn=0.392 and a Sp=0.821 and a cutoff value of 4 levels. Time to complete Level 1 on the WBBT ROC curve analysis revealed it is fairly accurate in discriminating the true negative rate, those that do have a concussion, (AUC=0.637, 95% CI: 0.542 to 0.732; p=0.007) with an associated Sn=0.875 and a Sp=0.253 with a cutoff value of 4.4 seconds. Among those who successful completed L5 of the WBBT, it is fairly accurate in discriminating the true negative rate, those that do have a concussion, (AUC = 0.60, 95% CI: 0.502 to 0.694; *p =* 0.05) with an associated Sn = 0.804 and an Sp = 0.392.

## 4. Discussion

The purpose of this study was to compare the performance of CONC to NORM on the WBBT. It was the aim of this study is to evaluate the WBBT as a potential tool for detecting postural instability in athletes with concussions. Our hypotheses were partially supported in this study. Athletes with Concussions and CONC did not demonstrate differences in the TT to complete the WBBT (CONC = 29.15 seconds, NORM = 28.79 seconds), yet they did complete significantly fewer Levels (CONC = 3.87 Levels, NORM = 4.18 Levels) of the WBBT. Furthermore, TL of the WBBT was fairly accurate in ruling out a concussive state (Sp=0.821). Additionally, CONC spent more time on L1 of the WBBT when compared to NORM and was fairly accurate at determine if one was concussed (Sn = 0.875). This finding is further supported by the post-hoc Chi-Square analysis that determine a significant relationship between the proportions of those who completed L5 between groups (Table 1). These findings could suggest a potential ceiling effect of the variable TT on WBBT. The investigation of the specific levels, completed and time, appear to be more accurate measures of postural performance on the WBBT when concussed. These findings suggest that CONC have a greater difficulty completing the L1 and L5 of the WBBT. However, with the AUC values falling within the fair to poor range for all statistically significant variables this test should be used to aid in the diagnosis process and not as a standalone assessment too.

From a postural control perspective, the WBBT demonstrates Fitts’s law of accuracy versus time to complete a series of goals [19]. Levels 1-4 increase in difficulty as demonstrated by the increased trend of time to completion for each group. These results could indicate that lower Levels of WBBT are easier to complete given the wider width of the target area. Thus a lower motor skill is relied upon and is appropriate for Levels 1-4 in healthy young adults. However, during L1 CONC took significantly longer time to complete than the CONC. This could indicate a potential pathology in either the feedback mechanism of postural control and/or a visual processing speed interference required for the CONC to adjust to the initial demands of the task [15]. Although this finding is not measured by common CoP metrics to approximate postural sway magnitude, a full second delay on completion of L1, along with its corresponding high Sn value, could be considered clinically relevant and still point to an abnormality within the postural control system. However, with the low Sp value (0.253) limits this variables potential clinical utility.

Conversely to the proposed pattern, L5 of the WBBT demonstrates a more challenging motor task in both groups. The target area is considerably smaller than all previous trials and could require a higher level of motor skill accuracy to complete the task. This is demonstrated by a smaller cohort of each group being able to complete L5 along with its corresponding Sn value (0.804). However, it should be noted that the proportion of CONC who completed L5 was significantly less when compared to the NORM group (Figure 3). This data suggests that NORM are able to complete L5 with more efficiency than CONC. This could also point to a potential pathology within the postural control system in CONC similar to time to complete L1.

No research has examined the WBBT in CONC nor does any study provide a systematic breakdown of the WBBT Levels of play. Research regarding quiet upright stance has demonstrated significantly higher sway magnitude in CONC within 24-48 hours post-injury [4,7,8,20,21]. Each of these studies relied upon varying methods and metrics of CoP to approximate sway magnitude and the flexibility of the motor system post- zinjury. Yet all of the studies used expensive research-grade equipment and sophisticated mathematical algorithms to determine postural instability. Although no CoP metrics were evaluated in this study, the reliability and validity of the Wii Balance Board approximation of CoP is similar to a research- grade force platform [9-11]. As such, the WBBT could be used as lower technology device to examine postural instability in post-concussion athletes, however this finding is purely speculative given the lack concurrent CoP data.

### 4.1. Clinical recommendations

Based upon the results of this study, if athletes with concussions take approximately a full second longer to complete LI and cannot complete L5 of the WBBT, they may have a postural dysfunction. Clinical use of these two values could aid in making objective decisions regarding postural instability post-concussion. This database can be used as a starting point for future research to compare an individual’s WBBT score post-concussion to determine if a potential balance dysfunction is present. However, without a reliable change index similar data to this study should not be compared to this database. Although this is limiting, it is the first database reported for the WBBT or any other Wii Fit game in an athletic post-concussed population. Furthermore, baseline data is considered the gold standard of assessment and comparison post-concussion.^1^ It is advised that baseline scores of the WBBT be used if possible for comparison if available.

### 4.2. Limitations

There are several limitations to this study. The lack of a direct comparison to other clinical postural assessment tools or research grade force platforms was not performed in this study. By potentially pairing the WBBT with other established clinical postural assessments, greater insight is expected. Due to the nature of concussion, the specific clinical data was unavailable for the CONC participants. Not knowing the specific symptoms or mechanism of injury could limit the findings of this study. Lastly, the WBBT is the first commercially available game capable of objectively measuring posture in an environmentally relevant context. However, without force plate data to examine the center of pressure movements during the assessment and with no reliable change index values, the WBBT should be used cautiously.

## 5. Conclusions

The current study examines the use of the Wii Balance Board Basic Balance Test in athletes with concussions. The results of this study suggest that the WBBT could be used as a novel objective low cost tool to aid in the diagnosis of postural instability within 24-48 hours post-injury. The provided data could aid health providers with an alternative method to measuring the presence of a postural deficit post-injury. Lastly, this normative database could be useful to clinicians in making return-to-play recommendations for collegiate athletes who experience a concussion and lack baseline balance data to compare their performance pre and post-injury.
